# Ototoxicity study in workers exposed to organophosphate

**DOI:** 10.1016/S1808-8694(15)30153-1

**Published:** 2015-10-18

**Authors:** Ana Cristina Hiromi Hoshino, Heloisa Pacheco-Ferreira, Carlos Kazuo Taguchi, Shiro Tomita, Maria de Fátima Miranda

**Affiliations:** a MSc, Speech and Hearing Therapist; b PhD, Adjunct Professor at the Medical School at UFRJ; c PhD, Adjunct Professor at Faculdade de Fonoaudiologia da Santa Casa SP; d PhD, Full Professor at the Medical School at UFRJ; e SPecialist, Assistant Professor at the Speech and Hearing Therapy School at UFRJ. Instituto de Estudos em Saúde Coletiva - IESC/UFRJ

**Keywords:** environmental exposure, intoxication, occupational risk, dizziness

## Abstract

Occupational and environmental exposure to agricultural pesticides represent an important health care problem in our country. Among the symptoms presented, dizziness stands out, because of a probable toxic action. **Aim**: The goal of our study was to characterize vestibular test results from rural workers occupationally and environmentally exposed to organophosphates used in agricultural pesticides. **Materials and Methods**: We carried out a cohort cross-sectional study with 18 rural workers from Teresópolis, RJ. Age ranged between 16 and 59 years, with a mean value of 39.6 years, 5 were males and 13 females. We used three questionnaires with questions associated with general and auditory health, and more specific questions about dizziness and its association with work. All workers underwent clinical evaluation, audiometry and vectoelectronystagmography. **Results**: results showed that 16 workers had irritative peripheral body balance disorder and 7 workers had sensorineural hearing loss, thus suggesting that agricultural pesticides cause vestibular alterations through a slow and silent intoxication. **Conclusions**: medical care for this population exposed to neurotoxic substances require an interdisciplinary approach, to guarantee efficient preventive and therapeutic measures.

## INTRODUCTION

Some chemicals have caused a lot of damage to human beings and the environment. Deterioration of forests, rivers, the air and impacts on human health have been observed as a result contact with such substances, either acute or gradual.

The increased presence of toxic substances has produced severe impact on the environment and the population exposed to their noxious effects. The use of pesticides, mainly in developing countries, is increasing every day. In Brazil, 5,570 cases of poisoning in an occupational context were reported in 2003, 1,748 (31.4%) by pesticide and 640 (11.5%) by other chemical products (Sistema Nacional de Informações Tóxico-Farmacológicas - SINITOX)^1^. Limited information, little education on the matter, and low control over production, distribution and use of these products are some of the main determining factors originating this relevant public health problem.

Exposure will often times produce silent clinical alterations that cannot be detected by biologic markers and that affect the lives of workers. Apart from the clinical effects observed in neurologic examination, subclinical symptoms can be assessed by means of neurophysiologic and neuropsychological tests^2^.

### Pesticides and ototoxicity

The US Environmental Protection Agency (EPA, 1980) defines pesticides as any substance or mixture of substances that aims to prevent, destroy, kill, or repel pests. Among the most broadly used in agriculture are anticholinesterase agents such as organophosphorous compounds that inhibit acetylcholinesterase (AChE), an enzyme that destroys the biologic activity of neurotransmitter acetylcholine (ACh). One it accumulates in the nerve ends (ACh), nerve electric stimulation does not stop. Toxicity signs include stimulation of muscarinic receptors of the parasympathetic system in the autonomous nervous system^3^.

Ototoxicity must be seen and studied as a matter of public health. Over 200 substances have been described in the literature as ototoxic, and most of them are not seen as noxious to men^4^.

For industrial toxicologists, ototoxic substances include all physical and chemical elements capable of damaging hearing. Jerger^4^ believes that ototoxicity is an undesired toxic response over the auditory and vestibular systems. Ototoxicity may be defined as the result of the action of certain drugs that damage the anterior and/or posterior labyrinth causing hearing loss with or without vertigo, nausea, or gait instability^5^.

Hearing loss may set in rapidly or more slowly, and severity depends on the amount, time of exposure, and interaction sustained with the ototoxic agent. Hearing loss may start during exposure or even months later, and is irreversible. It is mostly bilateral and symmetric, although cases of unilateral and asymmetric hearing loss have been reported. Vestibular involvement, although often concurrent, may appear earlier and more intensely than hearing symptoms.^6^

Many papers indicate that dizziness is a subjective symptom related to pesticide exposure.

Martin, Adams (2003)7 stress the importance of health care workers being aware of the symptoms connected to the use of chemical and biological weapons, so that quick and accurate diagnosis can be produced. Organophosphorous compounds, for instance, were characterized by their neurologic impacts and unspecific symptoms such as headache and dizziness.

Teixeira et al. (2003)^8^ carried out a study on prevalence with 98 workers with mean age of 41.6 years exposed to pesticides, organophosphorous compounds, and pyrethroids used in vector control campaigns. Rural workers were divided into two groups. Group 1 did not report exposure to noise in current and past occupational/leisure circumstances; group 2 reported exposure to noise in current and past occupational/leisure circumstances. The most frequent complaints were irritated eyes (54.1%), headache (44.9%), dizziness (35.7%), nausea, and torpor (16.3%). The most reported symptoms connected to nervous central system disorder were: difficulty understanding what others say (46%), difficulty remembering facts (43%), difficulty sustaining attention (24%), and hearing noises (24%). From those exposed only to insecticides, 63.8% had hearing loss; 66.7% of the group exposed concurrently to insecticides and noise had hearing loss. The authors concluded that there is evidence that exposure to pesticides induces peripheral auditory damage, and that noise is a factor that interacts with pesticides, thus enhancing ototoxic effects.

Delgado, Paumgartten (2004)9 interviewed 55 farmers on the use of pesticides through a questionnaire. They observed that 92% of the workers involved in handling these products had no personal protection equipment (PPE), 62% of them reported ‘feeling ill” while they handled pesticides. The most common symptoms were: headache, sickness, vomiting, vertigo, skin rash, and blurred vision.

The literature indicates that subclinical symptoms should be considered as early signs of poisoning^10^, ^11^.

Vestibular system alterations detected by tests are not reported in the literature. Therefore, only after vestibular examination according to the parameters defined for this study, will questions be answered or further research recommended.

## MATERIALS AND METHOD

This is a cross-sectional historical cohort study developed based on the vestibular function analysis of 18 rural workers from the municipality of Teresópolis, in the state of Rio de Janeiro, Brazil, located more specifically in the Córrego das Pedras and Baú regions. Ages varied between 16 and 59 years. Gender was not specifically targeted as a variable. Exposure time was calculated based on the number of years the workers have been active for.

Inclusion criteria were complaints of dizziness and occupational exposure to organophosphorous compounds; exclusion criteria were smoking, alcohol and drug abuse, metabolic, hormonal and neck disorders, as these factors are the main points where vestibular disorders are originated.

No specific age range was studied. The groups were not divided into age groups, as no data was found in the literature on worker age and normality patterns. Gender was not deemed as a relevant variable, as we followed the guidelines published by Mangabeira Albernaz, Ganança et al. (1986)^12^, in which differentiated normality patterns for each gender were not defined.

### Instruments and procedures

Three questionnaires were given to participants with the purpose of gathering information. They contained questions pertaining to the inclusion/exclusion criteria as specified previously.

Questionnaire I contained questions on ear anatomic and physiological disorders; auditory and vestibular symptoms; health in general, specifically addressing exclusion criteria and general information on the type of work they did. Questionnaires II and III contained questions on how work was done and its relations with dizziness.

All workers went through otorhinolaryngological, audiological, and vestibular examination.

In order to characterize normal hearing thresholds in adults, we adopted the hearing normality criteria for various ages defined by Glorig, Davis (1961)^13^ and Mangabeira Albernaz et al. (1981)^14^.

Vestibular examination was done using program Contronics under the principles defined by Mangabeira Albernaz, Ganança et al. (1986)^12^ both in terms of testing sequence and interpretation parameters.

### Ethical aspects

The study considered ethical aspects as recommended by Resolution 196/96 on research with human beings and included, among others, the obtention of free informed consent from participants. We ensured that individual participation would not lead to physical, psychical, moral, intellectual, social, cultural, or spiritual damage. This study was approved by our institution under permit 38/2005.

## RESULTS

### Age, gender, and time of exposure

Eighteen rural workers were interviewed. Most of them were women, as seen in Table 1. Mean age was 39.6 years, 55.6% of them were working for over 21 years, and 15 (83.3%) were working for 10 hours or more daily.

**Table 1:** Frequency of variables gender, age, years on the job, and working hours of 18 workers exposed to organophosphorous compounds, Teresópolis/RJ, 2006.

Variables	n	%
Gender		
Male	5	27,8
Female	13	72,2
Age		
16-35 years	8	44,4
36- 45 years	3	16,7
46-59 years	7	38,9
Years on the job		
0-10 years	4	22,2
11-20 years	4	22,2
21-45 years	10	55,6
Daily work hours		
<10 hours	3	16,7
>10 hours	15	83,3

### Used pesticides

The most broadly used pesticides were Manzate (54.2%) - toxicological rating III, followed by Folidol (33.3%), Tamaron (27.1%), and Gramoxone (25%), all under toxicological rating I, considered highly toxic (Decree 98.816/90 from Law 7802/89).

### Audiometric assessment

Audiometric tests found that 11 (61.14%) subjects had normal hearing, 7 (38.8%) had altered tone audiometry test results - 4 (22.22%) subjects had decreased performance at 6000Hz and 8000Hz and 3 (16.67%) had sensorineural hearing loss. All subjects had speech audiometry within normal ranges.

Seven subjects (38.9%) with normal tone audiometry tests were aged between 16-35 years and five subjects with altered tone audiometry test results (27,8%) were aged between 46-59 years.

Only two subjects (11.11%) had bilateral hearing loss.

### Vector electronystagmography (VENG)

Eighteen subjects underwent VENG, and 16 (88.8%) were found to have peripheral irritative vestibular syndrome, 11 of whom due to altered nystagmus test results and/or positional vertigo and 9 due to altered results in the caloric test, presenting hyperreflexia in absolute values as seen in Table 2.

**Table 2:** Description of types of alteration found under vector electronystagmography for 18 workers exposed to organophosphorous compounds, Teresópolis/RJ, 2006.

	NVE	PIVS			TOTAL
		PN	Hyperreflexia	PN + Hyperreflexia	
N	2	7	5	4	18
%	11,1	38,9	27,8	22,2	100

NVE Normal vestibular examination

Peripheral irritative vestibular syndrome

Altered results only in the positional nystagmus test

HYPERREFLEXIA Hyperreflexia in caloric test absolute values

PN + HYPERREFLEXIA Altered results in positional nystagmus test and hyperreflexia in caloric test absolute values

In terms of the aspects connected to the daily lives of the workers, we can see on Table 3 that 7 subjects (38.9%) do not work when they feel dizzy and 27.8% with altered test results reported they will still work when they feel dizzy. Ten subjects (44.4% with altered and 11.1% with normal test results) claimed dizziness is worse while they are working.

**Table 3:** Relationship between vector electronystagmography and issued connected to the daily lives of 18 rural workers exposed to organophosphorous compounds. Teresópolis/RJ, 2006.

		N		N		N	
		NVE	%	PIVS	%	TOTAL	%
	Sometimes	0	0	1	5,55	1	5,55
	No	0	0	7	38,9	7	38,9
Do you work when you”re dizzy?	Not always	0	0	1	5,55	1	5,55
	Yes	2	11,1	5	27,8	7	38,9
	Don”t know	0	0	2	11,1	2	11,1
	TOTAL	2	11,1	16	88,9	18	100
	No	1	5,55	3	16,65	4	22,2
Does the chemical product affect your balance?	Yes	1	5,55	12	66,7	13	72,25
	Don”t know	0	0	1	5,55	1	5,55
	TOTAL	2	11,1	16	88,9	18	100
	Yes	2	11,1	14	77,8	16	88,9
Is this work harmful to your health?	No	0	0	1	5,55	1	5,55
	Don”t know	0	0	1	5,55	1	5,55
	TOTAL	2	11,1	16	88,9	18	100
	Monday	0	0	1	5,5	1	5,5
	Friday	0	0	6	33,3	6	33,3
Monday Friday	All week	2	11,1	6	33,3	8	44,4
	Don”t know	0	0	3	16,8	3	16,8
	TOTAL	2	11,1	16	88,9	18	100
	When he or she wakes up	0	0	2	11,1	2	11,1
	At work	2	11,1	8	44,5	10	55,6
Morning evening	After work	0	0	1	5,5	1	5,5
	Always	0	0	3	16,7	3	16,7
	Don”t know	0	0	2	11,1	2	11,1
	TOTAL	2	11,1	16	88,9	18	100

NVE Normal vestibular examination

PIVS Peripheral irritative vestibular syndrome

Thirteen workers (72.25%) answered that the chemical products they use at work affect their balance, and 16 (88.9%) believe their work poses health risks. This last question did not directly relate hazardous working conditions to the use of pesticides.

Table 4 shows that 6 subjects (33.4%) with altered vector electronystagmography (VENG) test results were aged between 16-35 years and 7 subjects (38.9%) were aged between 46-59 years. Age did not interfere with test results. This finding is supported by the literature, as normality patterns are not stipulated for age groups.

**Table 4:** Correlation between vector electronystagmography tests and age range, years on the job, dialy working hours, and use of PPE in 18 workers exposed to organophosphorous compounds. Teresópolis/RJ, 2006.

		N		N		N	
		NVE	%	PIVS	%	TOTAL	%
	16-35 years	2	11,1	6	33,4	8	44,5
Age range	36-45 years	0	0	3	16,6	3	16,6
	46-59 years	0	0	7	38,9	7	38,9
	TOTAL	2	11,1	16	88,9	18	100
	0-10 years	0	0	4	22,2	4	22,2
Years on the job	11-20 years	0	0	4	22,2	4	22,2
	21-45 years	2	11,1	8	44,5	10	55,6
	TOTAL	2	11,1	16	88,9	18	100
	<10 hours	0	0	3	16,6	3	16,6
Working hours	>10 hours	2	11,1	13	72,3	15	83,4
	TOTAL	2	11,1	16	88,9	18	100
	Yes	0	0	2	11,1	2	11,1
PPE	No	2	11,1	14	77,8	16	88,9
	TOTAL	2	11,1	16	88,9	18	100

NVE Normal vestibular examination

PIVS Peripheral irritative vestibular syndrome

PPE Personal protection equipment

In contrast, the number of years on the job on the job and the daily time of exposure to pesticides interfered with test results, as 8 subjects (44.5%) with altered test results had been working for over 21 years and 13 subjects (72.3%) with altered VENG worked for more than 10 hours a day.

This scenario is worsened when we see that the use of personal protection equipment (PPE) could make a difference, as 77.8% of the subjects failed to use PPE and had altered vector electronystagmography results.

## DISCUSSION

Table 1 shows this is a quite young population and yet fully active from the labor standpoint, as most of them start working in farms from a very young age, following the steps of their parents. This practice is more common during the sowing and harvesting seasons. Gomide (1999)^15^ has reported that small and medium farmers are somehow aware of the health risks inherent to the use of pesticides. And they also say that after a few years of using pesticides they need to hire younger people to do this job, as they no longer feel able to do it. In their own words, young people are hired at 17, but by 25 or 26 they are no longer able to keep on doing the job.

Faria et al. (2004)^16^ tried to build a profile of pesticide exposure and analyze the incidence of poisoning by these products. Looking at a group of 1,379 farmers, they found that the incidence of pesticide poisoning was of 2.2 episodes for every one-hundred exposed workers; no differences were found in relation to gender.

Audiometric findings indicate that noise and chemical poisoning induced hearing loss are very similar to presbycusis. Hearing loss differential diagnosis can be done only through careful interview and clinical examination. Diagnosis can be quite challenging, as causes for disease often overlap.

Most subjects with normal and altered test results had been doing this job for 21-45 years. This indicates that hearing health does not vary according to time of exposure to pesticides. The same trend was observed when we looked at audiometric tests and number of hours individuals worked per day.

Failure to wear PPE (personal protection equipment) such as masks, gloves, and proper apparel to handle pesticides may be harmful to hearing. Teixeira, Augusto et al. (2003)^8^ and Manjabosco, Morata et al. (2004)^17^ indicated that pesticides induce hearing loss and that disease is worsened with noise.

Castro et al. (2005)^18^ interviewed farmers in Cachoeiras de Macacu (RJ) on pesticide risk perception and application practices. Many of their findings coincided with ours, in both farmer working conditions and quality of life. Their analysis showed that 22.5% of the farmers reported previous pesticide poisoning, while insecticide Decis 25 CE and herbicide Gramoxone (both extremely toxic) were the most frequently used in the region. Eighty-five percent of the farmers did not wear PPE, 27.5% threw empty pesticide containers in rivers or in the woods, 60% were never trained on how to handle pesticides, and that 85% said they were never asked to produce an agronomist prescription to buy pesticides. Farmers were sorted into three risk perception categories: 70% perceived risk but kept on using pesticides; 27% did not perceive risk; 3% perceived risk and stopped using pesticides.

All 18 rural workers that participated in our study had dizziness spells, 7 (38.9%) had hearing loss and 16 (88.8%) had vestibular disorders. Whenever reliable data on the possible impact a harmful substance may have on the environment or upon human health is available, the principle of precaution^19^ must be adopted, whether or not there is scientific certainty.

Dizziness stood out in our study as one of the clinical manifestations connected to pesticide exposure that must be assessed and detected during clinical interviews with rural workers and properly considered by the health care teams in charge of serving rural communities.

Workers are aware of the dangers pesticides offer to their health and the environment. They must therefore be trained on how to adequately use pesticides and informed of other means of dealing with their crops without the aid of pesticides.

The rural population exposed to pesticides is not limited to farmers. It also includes their families who live inside plantation areas and local communities. More research should thus be conducted on pesticide exposure assessment and the risk associated with these products. The literature sees dizziness as a subclinical symptom of acute and long term pesticide poisoning, a condition that adversely affects workers and renders them unable to work and live well with their families, deteriorating both health and quality of life.

## CONCLUSION

Considering the mean time for which the workers assessed in this study were exposed to pesticides and excluding the various aspects that contribute or trigger dizziness spells such as age, metabolic and hormonal disorders, social habits, among others, the data gathered in this study indicate that organophosphorous pesticides promote disorders in the vestibular and hearing systems, apart from potentially increasing the risk of neuroototoxic events.

The effects organophosphorous pesticides have upon human health encompass a variety of signs and symptoms that go way beyond clinical visibility, and thus call for a complex approach to health care so as to ensure treatment and prevention in terms of environmental and personal care in all its spheres.
